# Photobiocidal Activity of TiO_2_/UHMWPE Composite Activated by Reduced Graphene Oxide under White Light

**DOI:** 10.1021/acs.nanolett.4c00939

**Published:** 2024-06-25

**Authors:** Sang Bin Jeong, Ki Joon Heo, Jae Hyun Yoo, Dong-Gi Kang, Leonardo Santoni, Caroline E. Knapp, Andreas Kafizas, Claire J. Carmalt, Ivan P. Parkin, Jae Hak Shin, Gi Byoung Hwang, Jae Hee Jung

**Affiliations:** † Department of Mechanical Engineering, Sejong University, Seoul 05006, Republic of Korea; ‡ Indoor Environment Center, Korea Testing Laboratory, Seoul 08389, Republic of Korea; § School of Mechanical Engineering, Chonnam National University, Gwangju 61186, Republic of Korea; ∥ Lab. M.0, Seoul 04799, Republic of Korea; ⊥ Department of Chemistry, University College London, London WC1H 0AJ, United Kingdom; # Department of Chemistry, Imperial College London, Molecular Science Research Hub, White City Campus, 80 Wood Lane, London W12 OBZ, United Kingdom

**Keywords:** titanium dioxide, reduced graphene oxide, antimicrobial surface, white light, photobiocidal

## Abstract

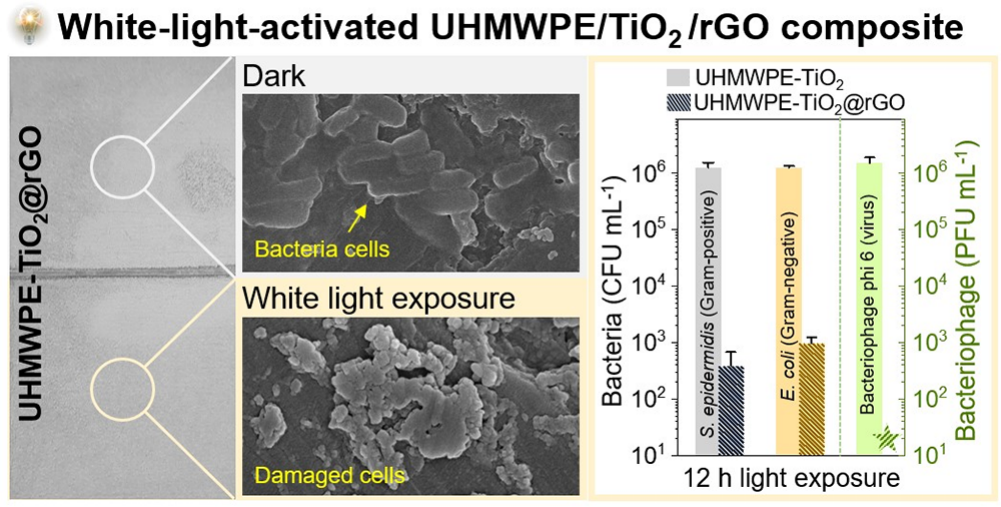

Herein, we introduce a photobiocidal surface activated by white light. The photobiocidal surface was produced through thermocompressing a mixture of titanium dioxide (TiO_2_), ultra-high-molecular-weight polyethylene (UHMWPE), and reduced graphene oxide (rGO) powders. A photobiocidal activity was not observed on UHMWPE-TiO_2_. However, UHMWPE-TiO_2_@rGO exhibited potent photobiocidal activity (>3-log reduction) against *Staphylococcus epidermidis* and *Escherichia coli* bacteria after a 12 h exposure to white light. The activity was even more potent against the phage phi 6 virus, a SARS-CoV-2 surrogate, with a >5-log reduction after 6 h exposure to white light. Our mechanistic studies showed that the UHMWPE-TiO_2_@rGO was activated only by UV light, which accounts for 0.31% of the light emitted by the white LED lamp, producing reactive oxygen species that are lethal to microbes. This indicates that adding rGO to UHMWPE-TiO_2_ triggered intense photobiocidal activity even at shallow UV flux levels.

Healthcare-associated infections (HAIs) pose a significant risk to vulnerable hospitalized patients including the elderly, infants, and people with a weakened immune system. HAIs are attributed to or associated with 7–10% of infection incidents in the world.^1^ According to the studies of Haque et al. (2018) and the World Health Organization (2011), it was estimated that approximately 100,000 and 37,000 patients died from HAIs in the US and Europe, respectively.^1,2^ A survey conducted in Canada also found that 8,000 people died from HAIs in 2013.^3^ Lydeamore et al. (2022) reported that 170,574 incidents of HAIs occur in adults admitted to public hospitals in Australia each year, resulting in 7,583 deaths.^4^ In addition, significant incidents of healthcare-associated SARS-CoV-2 (COVID-19) infection have been reported in recent years. It was estimated that up to 19.6% of patients with COVID-19 in UK hospitals became infected after hospital admission during the first pandemic wave and that 1–2% of all patients in England’s hospitals were infected by COVID-19 while being treated for other issues during the second wave.^5,6^ The disinfection of surfaces in hospitals is actively carried out to control HAIs. This is because surfaces can act as reservoirs for pathogens that are spread through frequent touch by healthcare workers and patients.^7−9^ With even minimal residual bacteria, some hospital surfaces remain consistently contaminated, quickly reproducing under favorable conditions and producing biofilms on surfaces, a source of pathogens that are spread in hospitals.

Photobiocidal surfaces have gained significant attention as a promising technique to inactivate hospital pathogens and keep surfaces sterile. Notably, the mechanism of pathogen inactivation from photobiocidal surfaces differs from antibiotic agents.^10−12^ When the surfaces are exposed to a light source, they are excited and induce reactive oxygen species (ROS), lethal to pathogens.^10,11^ Titanium dioxide (TiO_2_) is the most extensively studied photocatalyst for photobiocidal applications because of its nontoxicity and low cost.^13−15^ However, the application of TiO_2_, an ultraviolet (UV)-active photocatalyst, is restricted in a hospital setting because the lighting widely used in hospitals mainly contains visible light.^16,17^ Various strategies to enhance the photobiocidal activity of TiO_2_ surfaces under visible light have been suggested. Previous studies have introduced doping with metal nanoparticles (such as copper, silver, and platinum) onto TiO_2_. These nanoparticles, when excited by visible light, could enhance the photocatalytic reaction through light scattering, hot electron injection, and plasmon-induced resonance energy transfer.^18,19^ Studies that utilized visible light-activated organic dyes, such as crystal violet, methylene blue, and toluidine blue O, which are nonmetallic, were also explored.^20,21^ Electrons excited by visible light in the dyes induced advanced photocatalytic reactions, leading to strong photobiocidal activity.^20,21^ Carbonaceous materials have also been used to extend the photocatalytic activity of TiO_2_ into the visible light region. Among the various carbonaceous materials, graphene has received increasing attention. Graphene exhibits superb electron acceptor and transport properties and high chemical stability and can inhibit the recombination of photoinduced electron–hole pairs on the composite with TiO_2_,^22^ thereby promoting electron transport and photocatalytic efficiency. Accordingly, many studies have highlighted the application of graphene, graphene oxide (GO), or reduced graphene oxide (rGO) composites in TiO_2_-based photocatalytic research.^23−25^ In particular, rGO has been considered a promising catalytic material and widely used for the research of graphene-based semiconductor photocatalysts because rGO is relatively cheap and easy to produce on a large scale compared to graphene.^26−28^

Here, we introduce a white-light-activated biocidal surface (UHMWPE-TiO_2_@rGO) consisting of ultra-high-molecular-weight polyethylene (UHMWPE), anatase (TiO_2_), and rGO. The addition of rGO activated potent photobiocidal activity under a white LED light source. UHMWPE-TiO_2_@rGO showed potent photobiocidal activities against bacteria. It was even more significant against a SARS-CoV-2 surrogate virus. A comprehensive understanding of the photobiocidal activity of UHMWPE-TiO_2_@rGO was obtained through various mechanistic analyses. Scavenger/quencher assays showed that the combination of TiO_2_ and rGO induced the generation of the superoxide radical (O_2_^–^), hydrogen peroxide (H_2_O_2_), hydroxyl radical (^**·**^OH), and singlet oxygen (^1^O_2_). It also showed that the contribution of ^**·**^OH for microbe killing was higher than other ROS. Transient absorption spectroscopy (TAS) demonstrated that rGO in UHMWPE-TiO_2_ mainly enhanced the reaction of holes (as charge carriers) in the composite, which can promote the formation of ^**·**^OH and increase photobiocidal activity. In addition, TAS with electron scavengers showed that the photobiocidal activity was not induced by visible light but by the very low UV flux in the white light source.

Figure 1a shows the composite produced by the thermocompression process. The mixtures of UHMWPE and TiO_2_ (UHMWPE-TiO_2_) or UHMWPE, TiO_2_, and GO were loaded into a Teflon-coated aluminum mold and then compressed at 10 MPa and 200 °C for 1 h, producing composites 100 × 100 × 10 mm in size. We hypothesize that GO within the composite is converted into rGO during thermocompression, as heating at 200 °C is sufficient to remove oxygen-containing groups, including carboxyl, hydroxyl, and carbonyl. To determine the conversion, GO powder was exposed to heat at 200 °C, identical to that of thermocompression. Figure 1b and 1c show the conversion from GO to rGO before and after heat treatment at 200 °C. High-resolution XPS analysis of C 1s showed that the spectrum of GO was deconvoluted into three peaks at binding energies of 284.8, 287.2, and 288.9 eV, corresponding to sp^2^ carbon (C–C/C=C), epoxide (C–O), and carboxyl groups (O–C=O), respectively.^29,30^ The spectrum of the heat-treated GO showed that the intensity of the epoxide and carboxyl functional groups significantly decreased,^31^ indicating the removal of oxygen-containing groups by the heat treatment and causing conversion into rGO. TEM analysis showed that GO possessed a flat nanosheet structure. After the heat treatment, it significantly wrinkled, which we speculate was caused by the thermal evaporation of epoxide and carboxyl functional groups. UV–vis absorbance spectroscopy showed that absorption across the visible region increased upon conversion from GO to rGO (Figure S1). Thus, it is concluded that thermocompression on the mixture of UHMWPE, TiO_2_, and GO resulted in the formation of a UHMWPE-TiO_2_@rGO composite.

**Figure 1:**
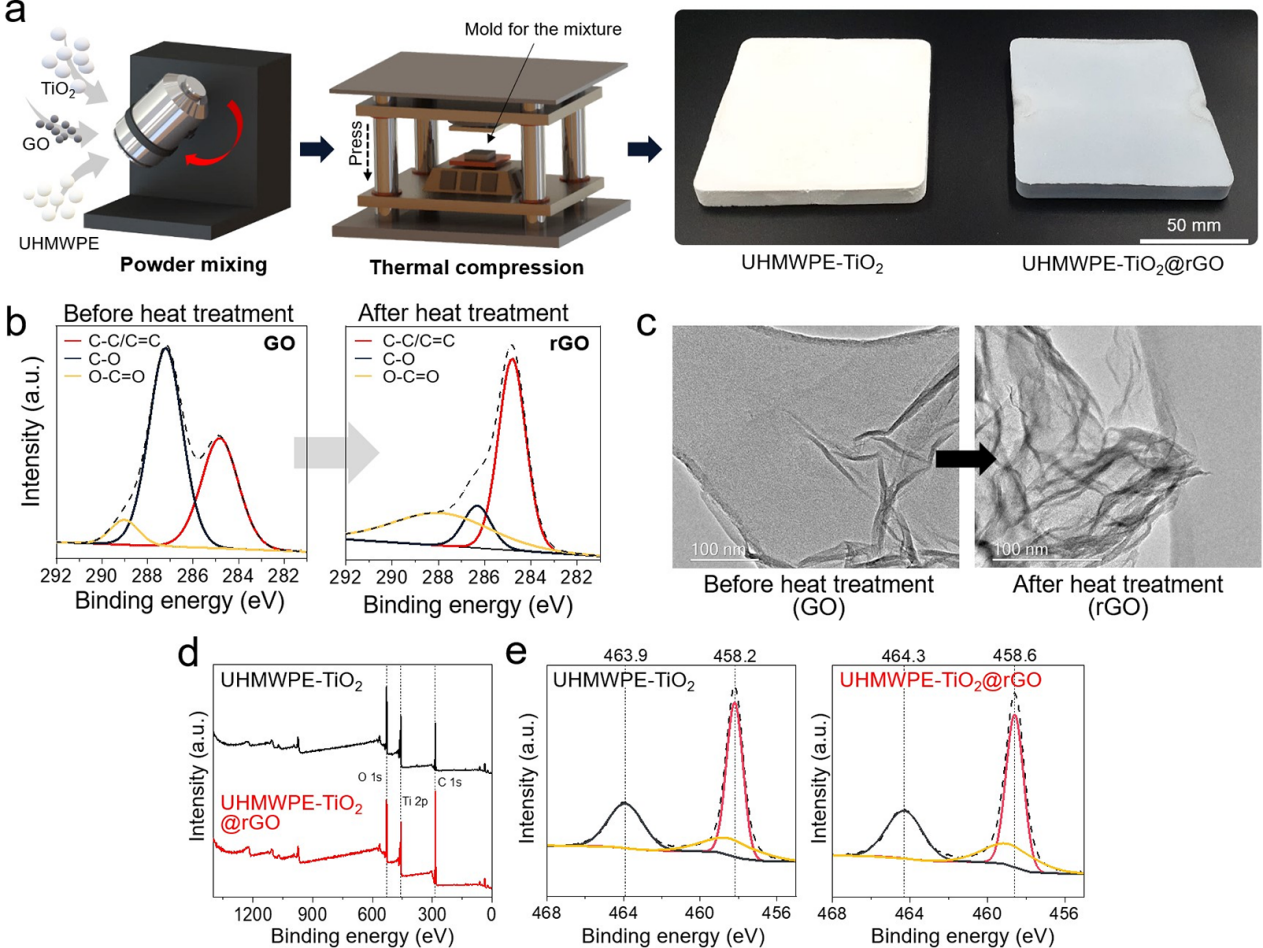
Characterization of the photobiocidal surface. (a) A schematic diagram for UHMWPE-TiO_2_ and UHMWPE-TiO_2_@rGO preparation. (b) High-resolution XPS spectra of C 1s of GO before and after exposure to heat at 200 °C for 1 h. (c) TEM image of GO before and after heat exposure. After the thermal treatment, graphene oxide (GO) was converted to reduced graphene oxide (rGO). (d) XPS survey spectra of UHMWPE-TiO_2_ and UHMWPE-TiO_2_@rGO. (e) High-resolution XPS spectra of the Ti 2p environment of UHMWPE-TiO_2_ and UHMWPE-TiO_2_@rGO.

Figure 1d shows the XPS survey spectra of UHMWPE-TiO_2_ and UHMWPE-TiO_2_@rGO. Ti and O peaks for TiO_2_ were observed in all samples, and the C peak showed the presence of UHMWPE, an extremely long chain of carbon-based monomers, alongside some adventitious carbon. The atomic percentage (at. %) for carbon in UHMWPE-TiO_2_@rGO was observed to be 5% higher than that in UHMWPE-TiO_2_. The high-resolution spectrum of Ti 2p for UHMWPE-TiO_2_ shows a double peak at binding energies of 458.2 and 463.9 eV for the 2p_1/2_ and 2p_3/2_ environments, indicative of Ti^4+^ states in TiO_2_.^32^ In the case of UHMWPE-TiO_2_@rGO, the binding energies of the double peak were 0.4 eV higher than those of UHMWPE-TiO_2_ (Figure 1e). This might be attributed to interactions of Ti and the O centers in rGO, where highly electromagnetic oxygen could decrease the electron density from Ti, resulting in a binding energy increase. In addition, diffusion reflectance spectra of UHMWPE-TiO_2_ and UHMWPE-TiO_2_@rGO at wavelengths of 250 to 750 nm showed that both samples had a main absorption at <350 nm (Figure S2). The light absorption of UHMWPE-TiO_2_@rGO at >350 nm was higher than that of UHMWPE-TiO_2_, indicating that the addition of rGO into UHMWPE-TiO_2_ enhanced its absorption of visible light.

A white LED lamp was used for the photobiocidal test. The white lamp mainly emitted visible light ranging from 380 to 650 nm with a small amount of UV light (200–380 nm; Figure 2a). The light intensity exposed to bacteria during the biocidal test was about 9.5 mW cm^–2^. Figure 2b shows the biocidal activity of UHMWPE only, UHMWPE-TiO_2_, and UHMWPE-TiO_2_@rGO against *S. epidermidis* in a dark room and under white light irradiation. A significant reduction in the number of viable bacteria was not observed on all tested samples after 12 h of exposure in the dark room. Upon 12 h exposure to white light, a viable bacteria reduction was not observed on UHMWPE only and UHMWPE-TiO_2_. In contrast, the reductions were observed on UHMWPE-TiO_2_@rGO, where the bacterial reduction increased with increasing rGO concentrations in the composite. The composite containing 1 wt % rGO showed a 3.6-log reduction of viable bacteria, where UHMWPE only and UHMWPE-TiO_2_ showed no significant reduction after 12 h exposure to white light. In further biocidal testing, the composite containing 1 wt % rGO was used. Figure 2c shows the biocidal activity of UHMWPE-TiO_2_@rGO with increasing exposure time in a dark room and white light. A minor natural decay of viable bacteria was observed on the surface of UHMWPE-TiO_2_@rGO with an increasing exposure time in the dark. Upon white light irradiation, more significant reductions in viable bacteria were observed, which increased with exposure time. After 24 h of light irradiation, the reduction reached below the detection limit (<100 colony forming unit (CFU) mL^–1^), equivalent to a >5 log reduction in the number of viable bacteria.

**Figure 2:**
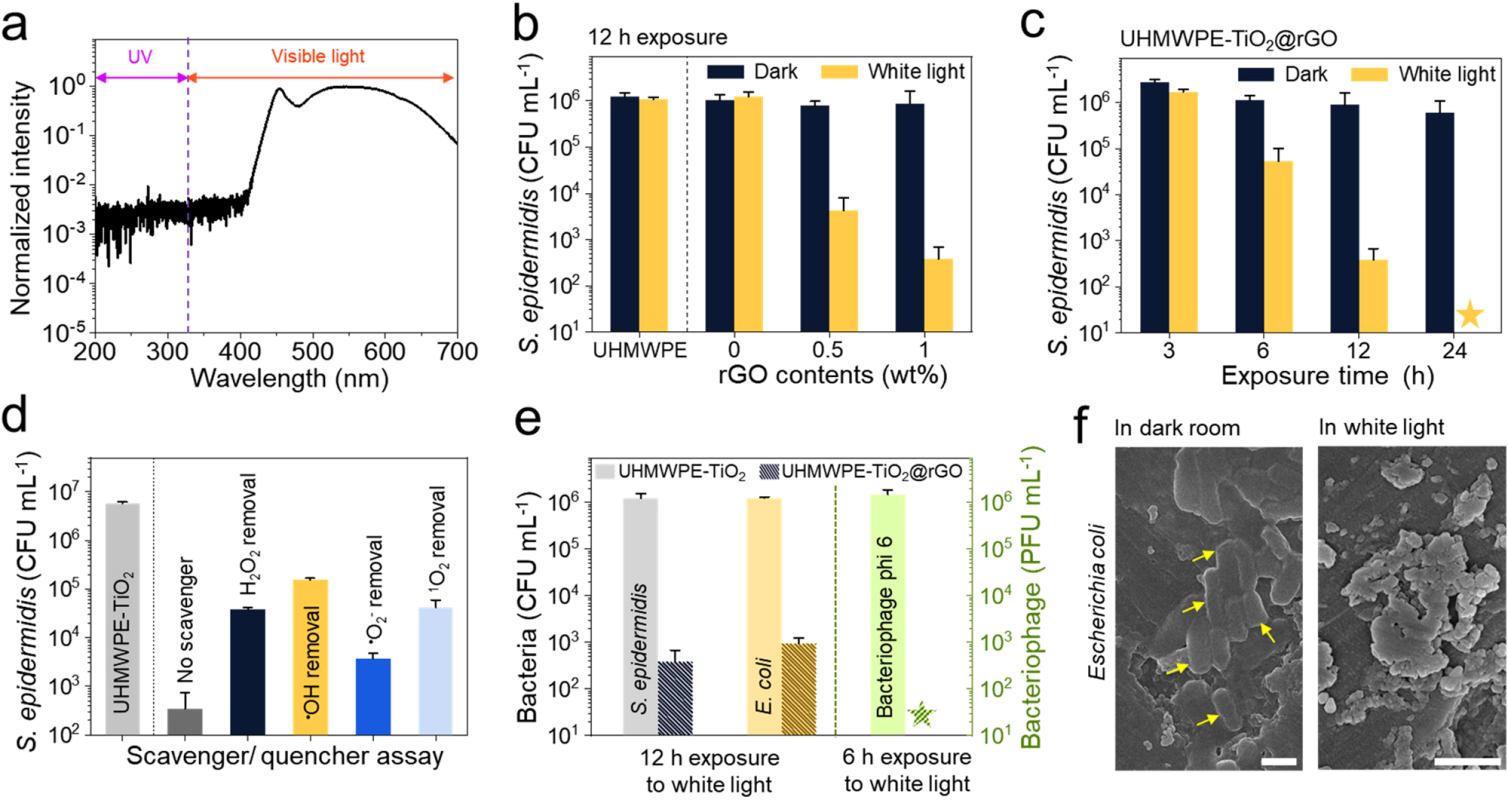
Photobiocidal activity of UHMWPE-TiO_2_ and UHMWPE-TiO_2_@rGO. (a) Light emission spectrum of the white light LED lamp used for the photobiocidal experiments. (b) Biocidal activity of pure UHMWPE, UHMWPE-TiO_2_, and UHMWPE-TiO_2_@rGO containing 0.5 or 1 wt % rGO in a dark room and white light. (c) Biocidal activity of UHMWPE-TiO_2_@rGO containing 1 wt % rGO with increasing exposure time in a dark room and white light. The star symbol indicates below the detection limit (100 CFU mL^–1^). (d) Scavenger studies of the biocidal activity of UHMWPE-TiO_2_@rGO containing 1 wt % rGO with the concomitant removal of H_2_O_2_, ^**·**^OH, O_2_^–^, and ^1^O_2_ in the respective presence of catalase, mannitol, superoxide dismutase (SOD), and l-histidine under white light LED irradiation. (e) Photobiocidal activity of UHMWPE-TiO_2_ and UHMWPE-TiO_2_@rGO containing 1 wt % rGO against *S. epidermidis*, *E. coli*, and bacteriophage phi 6. The star symbol indicates below the detection limit (100 PFU mL^–1^). (f) SEM images of the *E. coli* morphology after biocidal tests in the dark and in white light. Scale bars indicate 1 μm in length.

It is known that the photobiocidal mechanism of TiO_2_ starts with the absorption of light, resulting in the formation of electron–hole pairs. At the TiO_2_ surface, these electron–hole pairs can react with surrounding molecules, including the molecules O_2_ and H_2_O, to form ROS, including O_2_^–^ and ^**·**^OH, respectively. UHMWPE-TiO_2_ did not show any biocidal activity herein upon exposure to white light. However, the addition of rGO into UHMWPE-TiO_2_ activated potent photobiocidal activity. To determine the key ROS responsible for the photobiocidal effect observed on UHMWPE-TiO_2_@rGO, ROS scavenger and quencher assays were carried out. In the assay, photobiocidal activity was measured in the presence of scavengers (SOD, catalase, and mannitol) or a quencher (l-histidine). UHMWPE-TiO_2_@rGO exhibited potent photobiocidal activity without the addition of scavenger/quencher against *S. epidermidis*, with a 3-log reduction in viable bacteria number after 12 h of exposure to white light (Figure 2d). However, the addition of each scavenger or quencher reduced the photobiocidal activity, indicating that UHMWPE-TiO_2_@rGO induced generation of O_2_^–^, H_2_O_2_, ^**·**^OH, and ^1^O_2_, and each oxygen species played a role in the multisite attack on the bacteria. Of the oxygen species, the contribution of ^**·**^OH to the biocidal activity was higher than that of other species. After scavenging/quenching O_2_^–^, H_2_O_2_, and ^1^O_2_, the viable bacteria number was <4.2 × 10^4^ CFU mL^–1^, whereas after scavenging ^**·**^OH, it was >1.5 × 10^5^ CFU mL^–1^.

Phage phi 6 is an enveloped virus with size and morphological similarities to the SARS-CoV-2 virus, called Coronavirus.^33^ Thus, UHMWPE-TiO_2_@rGO were tested against *Escherichia coli*, Gram-negative bacteria, and phage phi 6, a SARS-CoV-2 surrogate, to determine its photobiocidal activity under white light irradiation (Figure 2e). A 3.2-log reduction in the number of viable *E. coli* bacteria was observed on UHMWPE-TiO_2_@rGO compared to UHMWPE-TiO_2_ after 12 h of exposure to white light. It has been reported that Gram-negative bacteria are more resistant to photobiocidal effects than Gram-positive bacteria because the bacteria have a more complex membrane structure.^34,35^ Thus, *E. coli* showed more resistance to the photobiocidal effect than *S. epidermidis.* Nevertheless, the potent photobiocidal activity of UHMWPE-TiO_2_@rGO was confirmed against Gram-positive and -negative bacteria. The reduction in the number of viable phage phi 6 viruses was more rapid on UHMWPE-TiO_2_@rGO than on the bacteria studied herein. The reduction reached below the detection limit (100 plaque-forming units (PFU) mL^–1^) after 6 h of exposure to white light, equivalent to a >5-log reduction of viable virus number. Bacteria can resist ROS attacks to some extent because they have antioxidant enzymes that can neutralize ROS and have internal systems that can repair cellular damage on their own.^36^ However, phage phi 6 does not have a cell membrane or cytoplasm that can physically resist ROS, and its capsid and envelopes are relatively vulnerable to ROS attack.^37,38^ Thus, the SARS-CoV-2 surrogate was more susceptible to ROS attack than were *S. epidermidis* and *E. coli*. Figure 2f shows the morphologies of *E. coli* after the biocidal tests in both dark and white light conditions. The bacterial morphology collapsed under white light irradiation, indicating the attack of ROS induced by UHMWPE-TiO2@rGO. This demonstrates that the ROS attack causes oxidative damage to DNA, protein, lipids, and the membrane of pathogens, resulting in cell death. In the case of *S. epidermidis*, a morphological collapse was not observed. This might be because the bacterial membrane has a rigid and thick peptidoglycan layer (detailed statement in Figure S3).^39,40^

TAS, which enables one to study the dynamic behavior of photogenerated charge carriers, was used to gain insight on the photobiocidal mechanism of UHMWPE-TiO_2_@rGO.^41,42^ Charge carrier generation was studied by using 355 and 532 nm laser excitation. A transient absorption signal was not detected in UHMWPE-TiO_2_@rGO at an excitation of 532 nm (Figure S4), indicating no generation of charge carriers at this visible wavelength. However, as shown in Figure 3a, strong signals were detected on UHMWPE-TiO_2_ and UHMWPE-TiO_2_@rGO at an excitation wavelength of 355 nm, indicating charge carrier generation at the UV wavelength. Importantly, no charge carrier generation on UHMWPE and UHMWPE@rGO was seen with any laser excitation (Figure S5), which showed that the TiO_2_ component was responsible for charge carrier formation. The shape of the decay of the transient absorption signal on UHMWPE-TiO_2_ and UHMWPE-TiO_2_@rGO was similar to previous reports of anatase TiO_2_, showing power law decay dynamics that can be attributed to the recombination of charge carriers via thermally driven trapping/detrapping processes.^41−43^ The signals on UHMWPE-TiO_2_ were stronger than those of UHMWPE-TiO_2_@rGO despite both samples having similar weight percentages of TiO_2_. This might be because the presence of rGO in UHMWPE-TiO_2_ may have parasitically absorbed UV light, preventing it from reaching TiO_2_ sites. Figure 3b shows the normalized decay kinetics at 800 nm for the composites. UHMWPE-TiO_2_ had higher transient absorption signals at 10 μs after the laser pulse than UHMWPE-TiO_2_@rGO. When the data were normalized, near identical decay behavior was observed in both samples, indicating that the addition of rGO to UHMWPE-TiO_2_ did not significantly alter the intrinsic charge carrier dynamics in this system. The reaction of photogenerated charge carriers on UHMWPE-TiO_2_ and UHMWPE-TiO_2_@rGO was investigated in the presence of electron (silver nitrate) and hole (methanol) scavengers.

**Figure 3:**
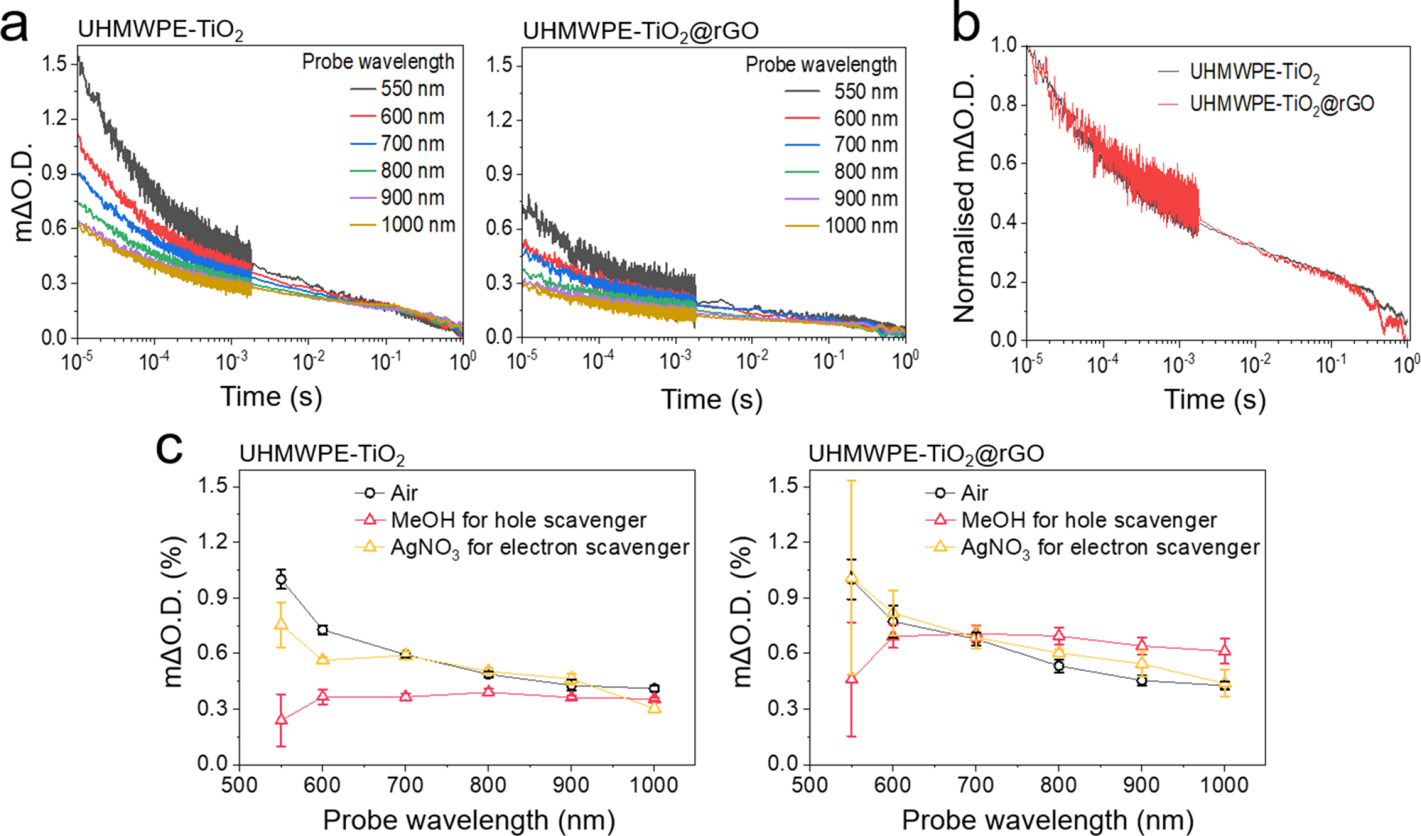
Photobiocidal mechanism of UHMWPE-TiO_2_@rGO: (a) Transient absorption decay kinetics for UHMWPE-TiO_2_ and UHMWPE-TiO_2_@rGO at selected probe wavelengths between 550 and 1000 nm (λ_exc_ = 355 nm, ∼6 ns pulse width, ∼0.10 mJ.cm^–2^, ∼0.67 Hz). (b) Normalized transient absorption decay kinetics for UHMWPE-TiO_2_ and UHMWPE-TiO_2_@rGO at 800 nm. (c) Normalized transient absorption spectra at 10 μs after the laser pulse in air, in methanol (which is a hole scavenger solution, MeOH), and in 50 mM aqueous silver nitrate (which is an electron scavenger solution, AgNO_3_), for both TiO_2_-UHMWPE and UHMWPE-TiO_2_@rGO samples (λ_exc_ = 355 nm, ∼6 ns pulse width, ∼0.10 mJ.cm^–2^, ∼0.67 Hz). For each sample, data have been normalized to the maximum transient absorption value observed in air.

Figure 3c shows the comparison of the scavenging efficiencies of the UHMWPE-TiO_2_ and UHMWPE-TiO_2_@rGO. Previous studies showed that in the presence of the electron scavenger, Ag metal particles form on the sample during the reaction, causing the lifetime and transient absorption of hole carriers to increase.^41,43^ For this electron scavenger, at early time scales, stronger transient absorption was seen in the blue region of the electromagnetic spectrum, with λ_max_ at ∼550 nm, and in the case of the hole scavenger, at early time scales, stronger transient absorption was seen in the red region of the electromagnetic spectrum, with λ_max_ at ∼700–800 nm.^43^ For UHMWPE-TiO_2_ and UHMWPE-TiO_2_@rGO, the ratios between the transient absorptions observed in air and the electron scavenger solution were similar, indicating that the electron scavenging efficiency was similar in both samples. However, the ratio between the absorptions observed in air and the hole scavenger solution was almost twice as large for UHMWPE-TiO_2_@rGO than that of UHMWPE-TiO_2_, indicating that holes on UHMWPE-TiO_2_@rGO were more reactive than UHMWPE-TiO_2_. The enhanced reactivity of holes can be related to ^**·**^OH generation and supports our ROS quenching/scavenger results (Figure 2d), showing that the enhanced photobactericidal activity is mainly due to ^**·**^OH formation. Previous studies reported that the combination of TiO_2_ and rGO created visible light-activated photocatalysts.^44,45^ However, most white light bulbs emit a small amount of UV light. Our TAS studies using 355 and 532 nm excitation showed that TiO_2_@rGO produced only charge carriers with 355 nm light, indicating that the photobiocidal activity of UHMWPE-TiO_2_@rGO could only be activated by UV light, accounting for 0.31% of light emitted by the white LED lamp rather than visible light. Thus, it is concluded that adding rGO into UHMWPE-TiO_2_ enhanced the ability of the composite to produce ^**·**^OH, acting as a cocatalyst that promoted the formation of this ROS more readily than the other composites studied herein, resulting in potent photobiocidal activity at very low flux levels of UV light.

In hospital settings, photobiocidal surfaces can be damaged under various extreme conditions, such as surface wear and fracture by friction and the impact of high-density materials. To determine its mechanical strength, UHMWPE-TiO_2_@rGO was tested in terms of impact strength and hardness. Figure 4a shows comparative data for gypsum, low-weight (LW) cement, high-strength (HS) cement, and UHMWPE-TiO_2_@rGO against impact stress. The impact strength was determined using an iron ball, weighing 265 g. UHMWPE-TiO_2_@rGO was fractured at an impact energy of 320.1 kJ m^–2^, whereas the gypsum, LW cement, and HS cement were fractured at 22.5, 22.5, and 66.7 kJ m^–2^, respectively, indicating that the impact strength of UHMWPE-TiO_2_@rGO was up 14.2 times stronger than other samples. As shown in Figure 4b, shore D hardness of UHMWPE-TiO_2_@rGO was measured using the ASTM D2240 standard method and compared to other rigid plastics, including high-density polyethylene (HDPE), thermoplastic polyurethane (TPU), and polypropylene (PP). The hardness of UHMWPE-TiO_2_@rGO could be categorized as extra hard with a shore D of 67, which is up to shore D 15 harder than other rigid plastics.^46−48^

**Figure 4:**
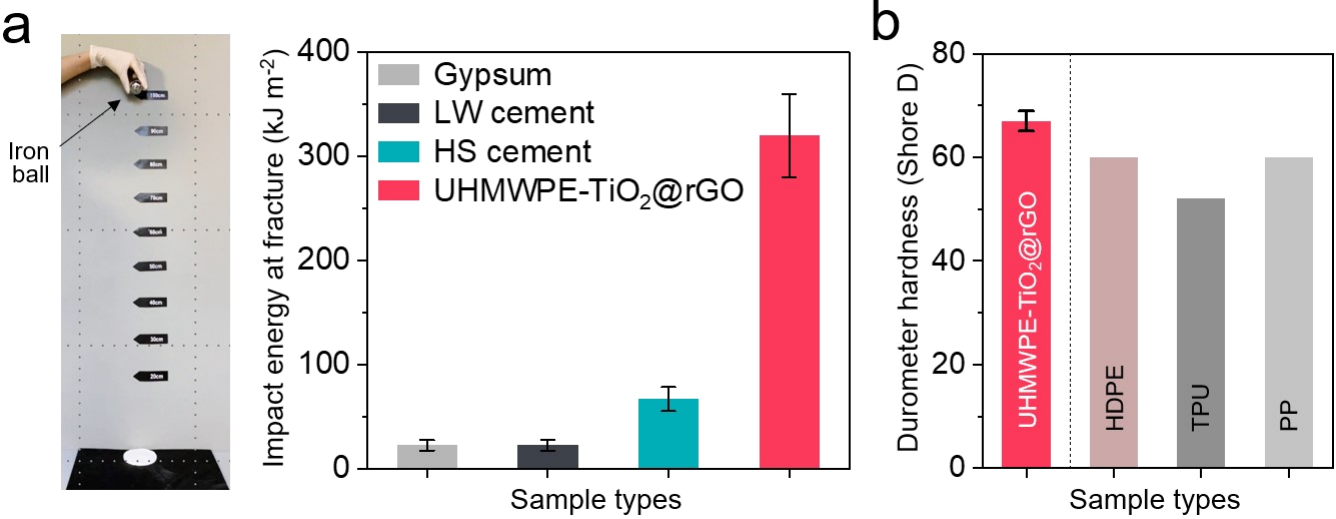
Mechanical properties of UHMWPE-TiO_2_@rGO. (a) Impact strength of gypsum, low-weight (LW) cement, high-strength (HS) cement, and UHMWPE-TiO_2_@rGO. (b) Durometer hardness of high-density polyethylene (HDPE), thermoplastic polyurethane (TPU), polypropylene (PP), and UHMWPE-TiO_2_@rGO. Shore D hardness data for HDPE, TPU, and PP was obtained from refs (46)–^48^.

This research introduced a white-light-activated photobiocidal surface composed of UHMWPE, TiO_2_, and rGO. The key findings of this research include (i) enhanced photobiocidal activity in white light, (ii) mechanism of rGO effect in photobiocidal activity, and (iii) mechanical durability and manufacturing compatibility.

First, testing under white LED lamps, commonly used in hospital settings, showed no photobiocidal activity for UHMWPE only and UHMWPE-TiO_2_. However, the addition of rGO to UHMWPE-TiO_2_ activated potent photobiocidal activities. After 12 h of white light exposure, UHMWPE-TiO_2_@rGO demonstrated a >3-log reduction of viable *S. epidermidis* and *E. coli* bacteria. The impact was even more pronounced against phage phi 6 virus, a SARS-CoV-2 surrogate with a >5-log reduction after 6 h of white light exposure. This highlights the practicality and effectiveness of UHMWPE-TiO_2_@rGO in killing a wide range of microbes, including Gram-positive, Gram-negative, and enveloped viruses. TAS showed that the UHMWPE-TiO_2_@rGO was likely activated by the portion of UV light emitted from the white light source, where rGO addition into the composite enhanced the reactivity of holes related to ^**·**^OH formation, acting as a cocatalyst that induced more ROS generation. Scavenger/quencher assays support the importance of these ^**·**^OH radicals in killing these pathogens compared to other species such as H_2_O_2_, O_2_^–^, and ^1^O_2_. Third, as demonstrated by mechanical experiments, UHMWPE-TiO_2_@rGO is highly resistant to external impact and friction. The thermocompressing process for fabricating UHMWPE-TiO_2_@rGO aligns with existing manufacturing methods for plastic-based products, allowing for seamless integration into industrial production.

Various techniques to produce biocidal surfaces have been suggested, with many showing satisfactory disinfection efficiency in laboratory settings.^16,49,50^ However, it is necessary for real-world applications to satisfy mass production while not reducing the biocidal performance.^51^ The simplicity of the mold-based thermocompressing process enables the efficient production of the biocidal surface in various shapes and sizes, tailored to specific needs. These advantages, coupled with the material’s scalability without sacrificing biocidal performance, make UHMWPE-TiO_2_@rGO a promising candidate for widespread application on hospital surfaces to prevent the spread of healthcare-associated infections.

## Supporting Information Available

Test procedure, UV–vis absorbance spectra, diffuse reflectance spectra, transient absorption decay kinetics, experimental details (PDF)
