# Supplementary material for: Photobiocidal Activity of TiO2/UHMWPE Composite Activated by Reduced Graphene Oxide under White Light
Source: Nano Lett. 2024 Jun 25;24(30):9155–62. doi: 10.1021/acs.nanolett.4c00939 (PMC11299222; doi:10.1021/acs.nanolett.4c00939)
Supplement: Supplementary file 1 — nl4c00939_si_001.pdf [file nl4c00939_si_001.pdf]

Supporting Information

Photobiocidal Activity of TiO_2/UHMWPE Composite Activated by Reduced Graphene Oxide under White Light

Sang Bin Jeong [a, b], Ki Joon Heo [c], Jae Hyun Yoo [d], Dong-Gi Kang [e], Leonardo Santoni [e], Caroline E. Knapp [e], Andreas Kafizas [f], Claire J. Carmalt [e], Ivan P. Parkin [e], Jae Hak Shin [a], Gi Byoung Hwang [e], Jae Hee Jung [a*]*

[a] Department of Mechanical Engineering, Sejong University, Seoul 05006, Republic of Korea

[b] Indoor Environment Center, Korea Testing Laboratory, Seoul 08389, Republic of Korea

[c] School of Mechanical Engineering, Chonnam National University, Gwangju 61186, Republic of Korea

[d] Lab. M.0, Seoul 04799, Republic of Korea

[e] Department of Chemistry, University College London, London, WC1H 0AJ, United Kingdom

[f] Department of Chemistry, Imperial College London, Molecular Science Research Hub, White City Campus, 80 Wood Lane, London W12 OBZ, United Kingdom

[*]Corresponding authors
Gi Byoung Hwang -Email: gi-byoung.hwang.14@ucl.ac.uk
Jae Hee Jung - Email: jaehee@sejong.ac.kr

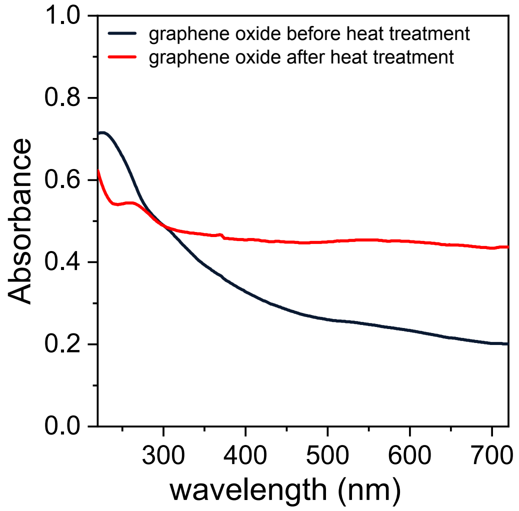

Figure S1. UV-vis absorbance spectra of graphene oxide before and after exposure to heat at 200°C for 1 h.

The UV-vis absorption spectrum of graphene oxide suspension showed a main absorbance at ~230 nm with a shoulder peak at ~300 nm. The peaks at ~230 and ~300 nm are attributed to π–π* transitions of the C–C aromatic bond and the n–π* transitions of the C=O bond, respectively. After 1 h exposure of graphene oxide to heat at 200 ^{o}C, the spectrum of graphene oxide showed a peak shift from ~230 to ~270 nm because of the removal of oxygen-containing groups and restoration of conjugated structures [1], indicating that after 1 h of heat treatment, the graphene oxide is converted into reduced graphene oxide. This trend is in agreement with that of previous research[2].

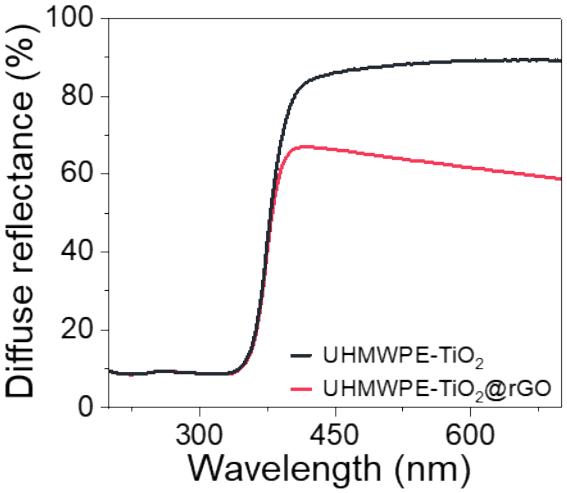

Figure S2. Diffuse reflectance spectra of UHMWPE-TiO_2 and UHMWPE-TiO_2@rGO, measured from 200 to 700 nm

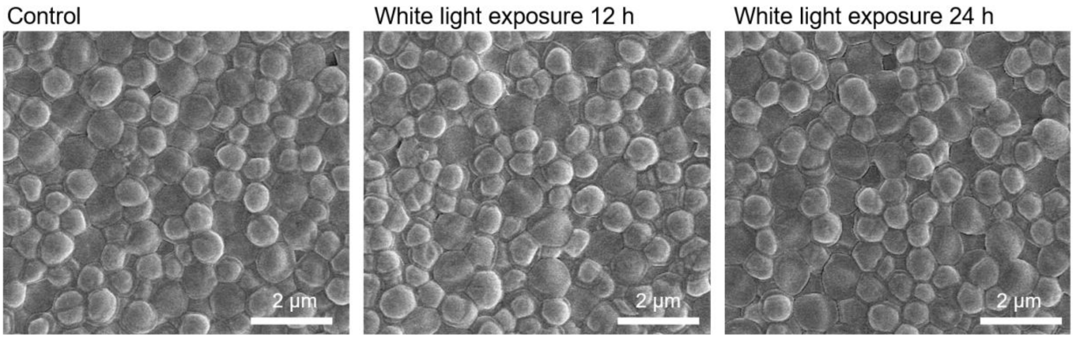

Figure S3. SEM images of the *S. epidermidis* morphology after photobiocidal test.

In the photobiocidal test, even after 24 h of white light exposure, the morphological damage of *S. epidermidis*, which is a Gram-positive bacterium, was not observed (Figure S3). It is speculated that the rigid and thick peptidoglycan layer in the bacterial membrane prevented the membrane from collapsing due to ROS attack[3-4]. The cell wall of the Gram-positive bacteria strain is made up of a plasma membrane, peptidoglycan whereas the wall of the Gram-negative bacteria strain has a more complex membrane consisting of plasma membrane, periplasmic space, peptidoglycan, outer membrane (lipopolysaccharide and protein)[4]. Thus, the cell wall of Gram-positive bacteria is more permeable to biocidal agents than Gram-negative bacteria[5]. It is speculated the ROS attack on *S. epidermidis* dominantly causes oxidative damage to DNA, RNA, protein, and lipids within the cell rather than the cell membrane.

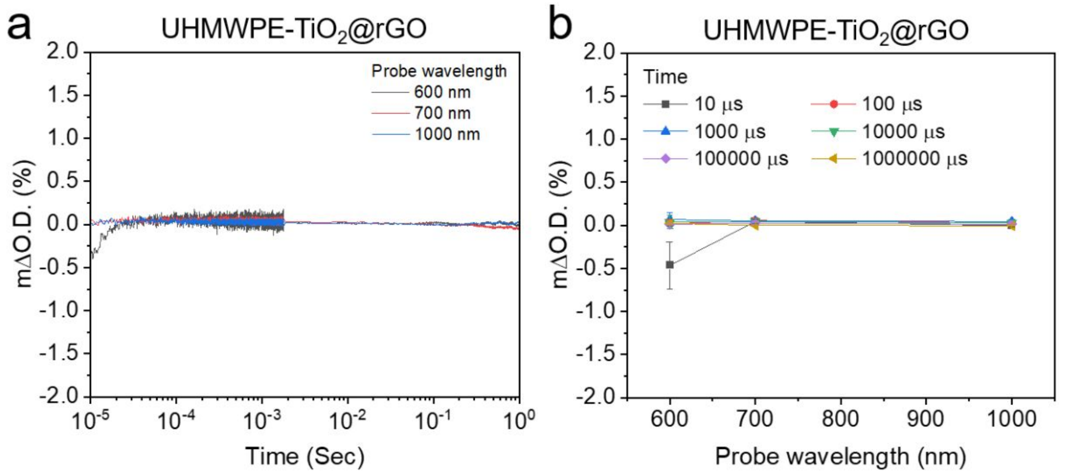

Figure S4. (a) Transient absorption decay kinetics at select probe wavelengths between 600 and 1000 nm, measured from 10 μs after the laser pulse until 1 s, and (b) transient absorption spectra at select times between 10 μs and 100 ms for UHWPE-TiO_2@rGO (λ_{exc} = 532 nm, ~6 ns pulse width, ~0.80 mJ cm^{-2}, ~0.67 Hz).

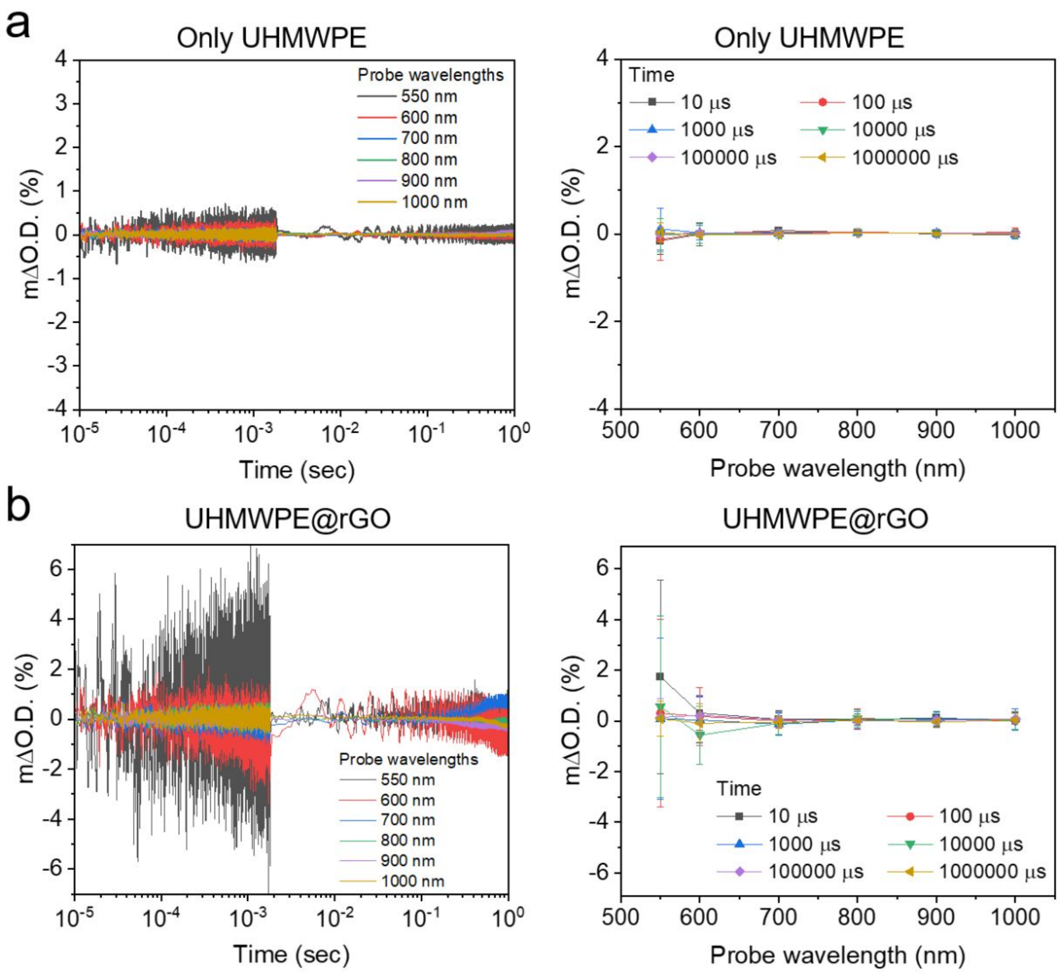

Figure S5. Transient absorption decay kinetics at select probe wavelengths between 550 and 1000 nm, measured from 10 μs after the laser pulse until 1 s (left), and transient absorption spectra at select times between 10 μs and 100 ms (right) for (a) only UHMWPE and (b) UHMWPE@rGO (λ_{exc} = 355 nm, ~6 ns pulse width, ~0.10 mJ cm^{-2}, ~0.67 Hz).

METHODS

Sample preparation

Anatase TiO_2 nanoparticles (99.5%, Sigma-Aldrich) were mixed with UHMWPE (Sigma-Aldrich) powder at a ratio of 1:1 in an electrical blender. Then, 0~1% of GO (Mzero Inc., Republic of Korea) powder was added to the mixture. UHMWPE was chosen as a binder for TiO_2 and GO due to its mechanical robustness and chemical stability. 120g of the mixture was placed into an aluminum mold coated by Teflon and thermally compressed at 200 ℃ for 1 h. The applied force to the mold was ~10 MPa. After that, the solid sample was collected from mold and cooled in a dark room for 24 h.

Bactericidal test

S. epidermidis (KCTC 1917; Korean Collection for Type Cultures, Republic of Korea) and *E. coli* (KCTC 1039) were incubated in nutrient broth (beef extract 0.3% and peptone 0.5%; Becton Dickinson, US) at 37℃ in a shaking incubator. When the bacterial medium reached an optical density of ~0.6 at 600 nm, the bacteria were harvested by centrifugation (4000 ×g, 15 min) and then washed out using deionized (DI) water. The titer of the bacterial suspensions was ~10^9 colony forming unit (CFU) mL^{-1} for both bacterial strains. For bactericidal tests, the suspension was 10-fold diluted using DI water to get ~ 10^8 CFU mL^{-1}. As shown in Figure S6, 40 µL of bacterial suspension was inoculated on the sample (20 mm × 20 mm), loaded on the rack, and then placed into a box with sterilized wet cotton. A sterile glass cover was placed on the top of the box to prevent moisture evaporation. The samples were exposed to white LED light (CLA60 9.5 W, 6500 K, AC 220 V/60 Hz; Osram GmbH, Germany) with an optical power of 9.5 mW cm^{-2} for a certain time, and another set of the samples was placed in a dark room. After exposure to LED light, the samples were placed into 10 mL of phosphate-buffered saline (PBS; Sigma Aldrich) and vortexed for 3 min to transfer bacteria from the sample to PBS. The bacterial suspension was serially diluted, plated onto a nutrient agar plate and incubated at 37 °C for 24 h. The bacterial colonies grown in the plate were counted after 24 h.

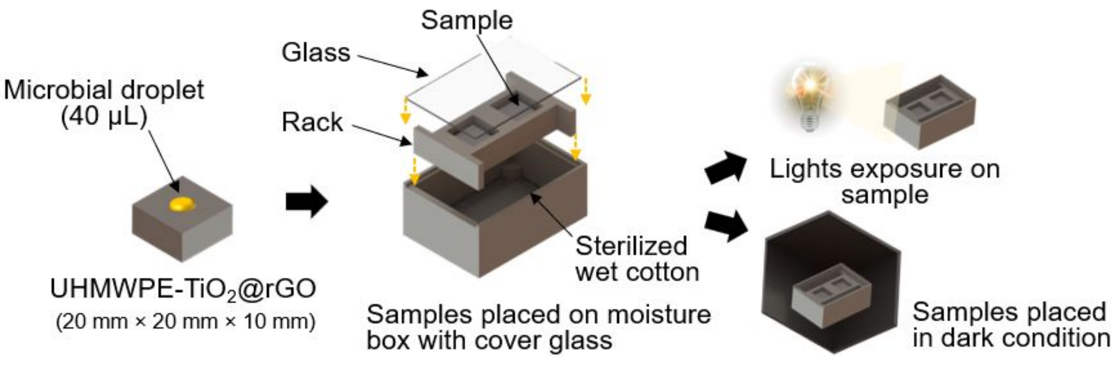

Figure S6. Test procedure of photobiocidal surface

Virucidal test

The host bacterium, *Pseudomonas syringae* (DSM 21482; DSMZ-German Collection of Microorganisms and Cell Cultures GmbH, Germany), was cultured at 25 ℃ in tryptic soy broth (TSB; Difco Laboratories, US) until an optical density reached ~0.3 at 600 nm. A single plaque of phage phi 6 (DSM 21518) was loaded in 100 μL of SM buffer (100 mM NaCl, 10 mM $MgSO_4$, 50 mM Tris-HCl [pH 7.5], and 0.01% [w/v] gelatin) to infect *P. syringae* cells. The infected cell medium was incubated at 25 ℃ in shaking with 170 rpm for 12 h. After centrifugation (4500 ×*g* for 20 min), the medium passed through a 0.2 μm filter (Minisart, Sartorius, Germany), and then the phage was collected using centrifugation (6000 ×*g*, 20 min) and stored at 4 ℃. A double-layer plaque assay was used to determine the concentration of phage phi 6 [51]. The double-layer plaque assay is explained as follows: Petri dishes with 15 mL of a bottom agar layer (TSB with 1.5% Bacto-Agar, Difco Laboratories) were prepared in advance. Top agar medium (TSB with 0.75% Bacto-Agar) was prepared in a water bath at 48℃. The host *P. syringae* (0.3 mL) and phage phi 6 (0.5 mL) were mixed with the top agar medium (14.2 mL) and poured on the bottom agar layer. Then, the double-layered agar plate was incubated at 25 ℃ until plaques became visible, and the plaques in the agar plate were counted. The bacteriophage titer was estimated to be ~10^9 plaque-forming units (PFU) mL^{-1}. The bacteriophage suspension was 10-fold diluted, and 40 μL of the suspension was inoculated on the sample. The sample with the rack was placed into the box with sterilized wet cotton and covered by sterile glass. The samples were exposed to white light from an LED for a certain time, and another set of samples was placed in a dark room. After the light exposure, the samples were placed into 10 mL of PBS solution (Sigma Aldrich) and vortexed for 3 min. Then, the number of viable phage phi 6 was determined using the double-layer plaque assay after a serial dilution.

Light source measurement

The spectra and optical power were measured under the condition that the distance between the light source and the sensor was 5 cm. This distance is the same as between the sample and the light source in the photobiocidal test. The sensor surface was covered with the same cover glass as in the photobiocidal test to accurately measure the light information reaching the sample. The spectrum of the white LED light (CLA60 9.5 W, 6500 K, AC 220 V/60 Hz; Osram GmbH, Germany) was measured using a spectrometer (USB2000+; Ocean Optics, Dunedin, FL, USA). In addition, an optical power was measured using a power meter (PM200; Thorlabs, Newton, NJ, USA). The optical power was 9.5 mW cm^{-2}, which was utilized for all photobiocidal performance evaluations.

Transient absorption spectroscopy

Transient absorption spectroscopy (TAS) was conducted in diffuse reflection mode to measure the charge carrier dynamics in samples from the microsecond to the second timescale. A Nd:YAG laser (Big Sky Laser Quantel Ultra, ~6 ns pulse width, 100 to 800 μJ cm^{-2}) operating at ~0.67 Hz was used as an excitation source, generating 355 nm UV light and 532 nm visible light from the third harmonic. The laser light was transmitted to the sample through a light guide. The probe light was a Bentham 610 quartz halogen lamp operated at ~100 W. Long-pass filters (Comar Instruments, UK) were placed between the lamp and sample to minimize short wavelength irradiation of the sample. Transient changes in diffuse reflectance from the sample were collected by a 2″ diameter, 2″ focal length lens and relayed to a monochromator (Oriel Cornerstone 130; Oriel Instrument, US) and measured at select wavelengths between 550 and 1000 nm. Time-resolved intensity data was collected with a Si photodiode (Hamamatsu S3071; Hamamatsu Photonics, Japan). Data at times faster than 10 ms was recorded by an oscilloscope (Tektronics DPO3012) after passing through an amplifier box (Costronics), whereas data slower than 10 ms was recorded on a National Instrument DAQ card (NI USB-6251; National Instruments, US). Each acquisition was triggered by a photodiode (Thorlabs DET10A; Thorlabs, Inc., US) exposed to laser scatter near the source. Data was acquired and processed by using home-built software written in Labview. Each kinetic decay represents an average of between 100 and 250 traces. Samples were measured in air or submerged in chemical scavengers, with methanol used as a photogenerated hole scavenger and aqueous silver nitrate solution (~50 mM) used as a photogenerated electron scavenger.

Scavenger/quencher assay

The scavenger/quencher assay determined key ROS responsible for photobiocidal activity. Catalase, *L*-histidine, mannitol, and superoxide dismutase (SOD) were purchased from Sigma-Aldrich. Catalase was used at a concentration of 6–14 units/mL in *S. epidermidis* suspension (~10^6 CFU mL^{-1}) to remove hydrogen peroxide (H_2O_2). Mannitol was used at a concentration of 82 mM in bacterial suspensions to eliminate hydroxyl radicals ($^{\bullet}OH$). SOD was used at a concentration of 20 units/mL in bacterial suspensions to remove superoxide radicals (O_2^-). *L*-histidine was used at a concentration of 2 mM in *S. epidermidis* suspension as a singlet oxygen ($^1O_2$) quencher. 6 mL of bacterial suspension, including ROS scavenger or quencher agents, was prepared in glass bowls. Then, the UHMWPE-TiO_2@rGO sample (20 mm × 20 mm × 10 mm) was submerged in each suspension and exposed to white light with an optical power of 9.5 mW cm^{-2} for 12 h. After that, the resulting suspension from each test was serially diluted, plated on a nutrient agar plate and incubated at 37°C for 24 h. The number of bacterial

colonies grown on the plate was counted.

Characterization using TEM, XPS, and SEM

High resolution-transmission electron microscope (HR-TEM; Titan G2 Cube 60-300, FEI Company, US) with 60 kV accelerating voltage was used to determine the morphology of graphene oxide before and after heat treatment at 200 ^{o}C. X-ray photoelectron spectroscopy (XPS, Thermo Fisher Scientific, US) analysis using a monochromatic Al-Kα X-ray source operated at 1486.6 eV was employed to obtain XPS data of graphene oxide, UHMWPE-TiO_2, and UHMWPE-TiO_2@rGO. All binding energies of the obtained peaks were referenced to the adventitious C 1s peak at 284.6 eV to compensate for surface charging effects. Field emission scanning electron microscopy (FE-SEM; Inspect F50, FEI Company, US) was used to observe *E. coli* cells on the surface of UHMWPE-TiO_2@rGO in dark and in white light. FE-SEM was operated under a high vacuum at 15 kV electron accelerating voltage.

Mechanical testing

Gypsum, low-weight (LW) cement and high-strength (HS) cement were prepared as comparison samples for impact test. The comparison samples were dried in a mold for 28 days to optimize their mechanical strength. The impact test was performed using an iron ball with a diameter of 30 mm and a weight of 265 g. For the experiment, samples with a thickness of 10 mm were prepared. The iron ball was vertically impacted onto the centre of the sample at a distance of 50 to 1,000 mm. The impact energy was calculated at the height where the sample was fractured. The gypsum and cement samples used for comparison were dried in molds for 28 days to optimize their mechanical strength. The hardness of UMHWPE-TiO_2@rGO was measured using the American Society for Testing and Materials (ASTM) D2240 standard. The hardness was measured using the Asker D-type durometer (Asker D, Asker Durometer, Kobunshi Keiki Co., Ltd., Japan).

References

(1) Gurunathan, S.; Han, J. W.; Eppakayala, V.; Kim, J. H. Microbial reduction of graphene oxide by Escherichia coli: a green chemistry approach, *Colloids Surf. B*, **102**, 772-777 (2013).

(2) Sunderrajan, S; Miranda, L.R.; Pennathur, G. Improved stability and catalytic activity of graphene oxide/chitosan hybrid beads loaded with porcine liver esterase, *Prep. Biochem. Biotechnol.*, **48**, 343-351 (2018).

(3) Silhavy, T. J.; Kahne, D.; Walker, S. The bacterial cell envelope, *Cold Spring Harb Perspect. Biol.*, **2**, a000414 (2010).

(4) Tortora, G. J.; Funke, B. R.; Case, C. L. Microbiology: an introduction; Pearson Bengamin Cummings, 1988.

(5) Fischer E.; Braun V. Permeability barrier of bacterial cell envelopes as cause of resistance to antibiotics. Immun Infekt., **9**,78-87(1981).
